# Is nodding syndrome in northern Uganda linked to consumption of mycotoxin contaminated food grains?

**DOI:** 10.1186/s13104-018-3774-y

**Published:** 2018-09-24

**Authors:** Richard Echodu, Hilary Edema, Geoffrey Maxwell Malinga, Adam Hendy, Robert Colebunders, Joyce Moriku Kaducu, Emilio Ovuga, Geert Haesaert

**Affiliations:** 1 Department of Biology, Faculty of Science, Gulu University, P.O. Box 166, Gulu, Uganda; 2 Gulu University Bioscience Research Laboratories, P.O. Box 166, Gulu, Uganda; 3 Department of Environmental and Biological Sciences, Faculty of Science and Forestry, University of Eastern Finland, P.O. Box 111, 80101 Joensuu, Finland; 4 Department of Pathology, University of Texas Medical Branch, Galveston, USA; 5 Global Health Institute, University of Antwerp, Antwerp, Belgium; 6 Department of Pediatrics, Faculty of Medicine, Gulu University, P.O. Box 166, Gulu, Uganda; 7 Department of Mental Health, Faculty of Medicine, Gulu University, P.O. Box 166, Gulu, Uganda; 8 Department of Plants and Crops, Faculty of Bioscience Engineering, Ghent University, Gent, Belgium

**Keywords:** Nodding syndrome, Mycotoxin, Food grain, Uganda

## Abstract

**Objective:**

Nodding syndrome (NS) is a type of epilepsy characterized by repeated head-nodding seizures that appear in previously healthy children between 3 and 18 years of age. In 2012, during a WHO International Meeting on NS in Kampala, Uganda, it was recommended that fungal contamination of foods should be investigated as a possible cause of the disease. We therefore aimed to assess whether consumption of fungal mycotoxins contributes to NS development.

**Results:**

We detected similar high levels of total aflatoxin and ochratoxin in mostly millet, sorghum, maize and groundnuts in both households with and without children with NS. Furthermore, there was no significant association between concentrations of total aflatoxin, ochratoxin and doxynivalenol and the presence of children with NS in households. In conclusion, our results show no supporting evidence for the association of NS with consumption of mycotoxins in contaminated foods.

**Electronic supplementary material:**

The online version of this article (10.1186/s13104-018-3774-y) contains supplementary material, which is available to authorized users.

## Introduction

Nodding syndrome (NS) is an epileptic disorder occurring among certain rural African populations in onchocerciasis endemic regions. It is characterized by repeated head-nodding seizures, developmental retardation and growth faltering. The first nodding seizures occur in previously healthy children between the ages of 3–18 years [1–3]. The disorder has been observed in onchocerciasis endemic regions in Uganda [3, 4], South Sudan [2] and Tanzania [5, 6], but children with similar symptoms were also described in other onchocerciasis endemic regions in Liberia [7] and Cameroon [8].

In northern Uganda (Acholiland), the first cases of NS were diagnosed retrospectively in Kitgum district around 1997 to 1998 and were later reported from the neighbouring districts of Lamwo and Pader [9]. NS remains a public health problem in Uganda where it is associated with high morbidity and mortality, severe socio-economic consequences, and social exclusion [1, 10].

Currently, the natural history, etiological agent and pathogenesis of NS remain unknown, and there is no specific treatment for those affected. Despite this, many children receive antiepileptic therapy which improves outcome [11]. However, for effective management and control of NS to take place, it is important that the causative agent of the disease is identified. The hypothesized causes so far have included infections with micro-organisms such as trypanosomes, malaria, measles, cysts or viruses [3], genetic epilepsy disorders [3, 11], chemical warfare; infectious agents [3, 12], infection with the filarial parasite *Onchocerca volvulus* [2, 3, 12, 13] and toxins and nutritional deficiencies [3, 12, 14]. However, the only associated risk factor observed in epidemiological studies in all regions where NS or NS-like symptoms have been reported is an *O. volvulus* infection. There is currently increasing evidence to support the hypothesis that NS is triggered by infection with *O. volvulus*, and that NS is only one in a spectrum of clinical presentations of onchocerciasis-associated epilepsy [15]. However, in 2012, during a WHO International Meeting on NS in Kampala, it was stated that fungal contamination of foods with mycotoxins required further study as a possible cause of NS [16].

So far little attention has been paid to the potential role of mycotoxins consumed in staple foods in causing neurological diseases despite their known neurotoxic effects in humans. Mycotoxins are secondary metabolites produced by toxigenic fungi that infect food crops both in fields and stored foods [17]. Recent studies have demonstrated that the *Fusarium* mycotoxin fumonisin B1, often present in maize, interacts with neuroblastoma cells leading to mitochondrial membrane potential depolarization and calcium deregulation [18]. More evidence shows that fumonisin B1 makes neurons more vulnerable to epileptiform conditions. The ribotoxin deoxynivalenol (DON) has been reported to interfere with protein biosynthesis through binding of ribosomal subunits. This affects brain homeostasis and possibly participates in the etiology of neurological diseases in which alteration of the glia are involved [19]. We investigated whether mycotoxins could be a co-factor in developing NS and determined the concentrations of mycotoxins in grain-based consumed staple foods in Northern Uganda in households with and without children with NS.

## Main text

### Methods

#### Study sites

The study was conducted in the districts of Kitgum (3°17′20.0″N, 32°0.52′40.0″E) and Lamwo (3°32′0″N, 32°48′0″E) in northern Uganda, bordering South Sudan. The total land area of the two districts is 9556 km^2^ characterized by woody savannah vegetation with a population of 338,427 [20–22]. These two districts were affected by civil war between the Lord’s Resistance Army (LRA) and the Uganda People’s Defense Force, which disrupted social service delivery between the mid 1980’s and 2006. It also resulted in the creation of many internally displaced person (IDP) camps. The majority of the population rely on small scale agriculture as a primary source of income [22]. Ninety percent of farmers are engaged in crop production, while a small percentage rear livestock, including Ankole and Zebu cattle in the Mid North [22, 23]. The northern districts receive around 750–1500 mm of annual rainfall [20]. The dry season, which lasts from November until March, can be severe. Drought tolerant crops are therefore cultivated and include finger millet, sesame, cassava and sorghum [23].

#### Recruitment of households

A total of 38 households with 62 children with NS cases, and 46 households with children without NS cases were recruited for the study (Additional file 1: Table S1). The diagnosis of NS was made by an experienced pediatrician (JMK) by medical history taking, clinical examination and if available, reviewing the medical records. NS was defined according to the WHO case definition of NS.

#### Collection of food grain samples

Collections of grain-based food samples from households with and without NS cases were made in seven villages (Additional file 2: Table S2) between November 2014 and July 2015. These samples were picked from storage bags in randomly selected households with or without NS (Additional file 1: Table S1). The samples included sorghum, maize, millet, and sesame. We collected 500 g of cereal grain per household and stored each sample separately in a polythene bag labeled with a unique identification number. In total, 105 cereal grain samples were collected from the two districts (Additional file 2: Table S2). These samples were transported to the Gulu University Bioscience Research Laboratory where mycotoxin was extracted, as described below, and stored at 4 °C until analysed.

#### Mycotoxin analysis

We carried out quantitative analyses of total aflatoxin, ochratoxin and DON on the grain-based food samples collected from all households. Briefly, 20 g of each grain sample was thoroughly ground in a blender (IKA, Model M20, Germany) and aflatoxin and ochratoxin were extracted using methanol, whereas distilled water was used for DON extraction (as per the Romer Labs kits procedures). Each sample was then filtered through fluted filter paper (Whatman 1; WHATMAN International Ltd, England) and the elute was used for analysis. The assays for total aflatoxin, ochratoxin and deoxynivalenol were performed on all the samples by direct competitive enzyme-linked immunosorbent assay (ELISA) using AgraQuant assay kits (Romer Labs Singapore Pte Ltd). Results were measured optically using an ELISA reader MULTISKAN FC, model 357, China) with an absorbance filter of 450 nm (OD450) and differential filter of 630 nm. Standard curves for the mycotoxins were generated from kit sample standards and inbuilt Log-logit regression models using Microsoft Excel 2013.

#### Statistical analysis

We examined differences in concentrations of aflatoxin, ochratoxin and DON in sampled grains between households in each study group using the Mann–Whitney U test. A Pearson correlation analysis was used to establish the relationship between concentrations of aflatoxin, ochratoxin and DON in sampled grains and the presence of children with NS in households. Statistical analyses were run with alpha set at 0.005 [24]. All analyses were conducted using IBM SPSS version 24 (Armonk, NY, USA).

#### Ethical consideration

This study was approved by the institutional review board of St. Mary’s Hospital Lacor (LHIREC 028/05/14). Formal approval to conduct the study was granted by the Uganda National Council for Science and Technology and the Office of the Ugandan president (HS1824). Heads of households provided written informed consent.

### Results

#### Concentrations of aflatoxin, ochratoxin and DON

The concentrations of total aflatoxin, ochratoxin and DON did not differ significantly between food grains sampled from households with and without children with NS (Table 1).

**Table 1:** Concentrations of total aflatoxin, ochratoxin and DON in sampled food grains in households with (n = 38) and without (n = 46) children with NS

Mycotoxin	Median (interquartile range)		Mann–Whitney U Test	*p* value
	Households with NS cases	Households without NS cases		
Total aflatoxin	4.3 (0–9.2)	3.8 (0–7.8)	795	0.47
Total ochratoxin	1.7 (0.7–3.0)	1.7 (0.4–3.4)	858	0.88
DON	0.1 (0–0.6)	0.01 (0–0.5)	833	0.70

#### Relationship between concentrations of mycotoxins in food grains and NS

There were no significant correlations between concentrations of total aflatoxin, total ochratoxin and DON in food grains sampled and the presence of NS in households in the two districts (Table 2).

**Table 2:** Pearson correlation coefficient matrix between concentrations of total aflatoxin, total ochratoxin and DON in sampled grains and cases of NS in Kitgum and Lamwo districts, Uganda

	Total aflatoxin	Total ochratoxin	DON	Household with children with NS cases
Total aflatoxin				
Total ochratoxin	− 0.419 (< 0.001)			
DON	− 0.171 (0.12)	− 0.064 (0.57)		
Household with children with NS cases	0.110 (0.32)	0.044 (0.69)	0.124 (0.26)	

Correlation coefficient r values are indicated (p-values in brackets)

### Discussion

We assessed the association between concentrations of mycotoxins in grain-based consumed staple foods in Northern Uganda in households with and without children with NS, with the aim to detect a potential role in disease development. The concentration of total aflatoxin, total ochratoxin and DON were high in households both with and without children with NS, without there being a statistical difference between the two groups. There appeared to be a stronger association between aflatoxin consumption and NS, than for both ochratoxin and DON, but this was not statistically significant (Table 2). This could suggest that aflatoxin can be a risk factor in hastening the development of NS considering its neurotoxic effect and neural tube defects [25].

Our study has one major limitation. Most children involved in this study developed NS many years before the study, when living in the IDP camps. The food children received in these camps were different from the food families were eating at the moment of the study and therefore we cannot exclude that it is possible that in these camps children who later developed NS were more exposed to contaminated food compared to controls. High levels of aflatoxin were detected in foods of households with and without children with NS. Daily consumption of small doses of aflatoxin can lead to its accumulation which could potentially be neurotoxic. Children are more sensitive to mycotoxins due to their high metabolic rates, low body mass, underdeveloped organ functions and detoxification mechanisms [26]. It is also possible that mycotoxin toxicity can act as a cofactor in NS disease development in certain individuals with genetic or immunological complications as has been suggested by Dowell [3] and Idro [11]. An association between stunted growth in children and high intake of aflatoxin was reported from Benin and Togo [27, 28].

Mycotoxin contamination is known to have profound health risks in certain populations, and high mortality rates have been recorded in countries including Kenya, India and Ethiopia [29–31]. There is greater risk of toxicity caused by human consumption of mycotoxins in northern Uganda due to chronic dietary exposure to contaminated food grains. Dietary aflatoxin exposure has been associated with human hepatocellular carcinomas particularly in areas of high hepatitis B prevalence such as in northern Uganda [32]. Most African countries remain at high risk of mycotoxin contamination, while children are much more susceptible to the effects of toxicity as has been shown in studies in Benin and Togo [27, 28].

### Conclusion

Our results show no supporting evidence for the association of NS with consumption of mycotoxins in contaminated foods.

## Limitations

We acknowledge the limitations of the current study including:

- Most children involved in this study developed NS many years before the study, when living in the IDP camps.
- The food children received in these camps were different from the food families were eating at the moment of the study and therefore we cannot exclude that it is possible that in these camps children who later developed NS were more exposed to contaminated food compared to controls.

## Additional files

**Additional file 1: Table S1.** Concentration of mycotoxins in grain samples from Lamwo and Kitgum in households with NS and without NS.

**Additional file 2: Table S2.** Sampling sites for cereal grains.

## Abbreviations

NS: nodding syndrome
DON: deoxynivalenol
ELISA: enzyme-linked immunosorbent assay
LRA: Lord’s Resistance Army
IDP: internally displaced person’s camps
OD: optical density
