# Supplementary material for: Is nodding syndrome in northern Uganda linked to consumption of mycotoxin contaminated food grains?
Source: BMC Res Notes. 2018 Sep 24;11:678. doi: 10.1186/s13104-018-3774-y (PMC6154931; doi:10.1186/s13104-018-3774-y)
Supplement: Supplementary file 1 — Additional file 1: Table S1. Concentration of mycotoxins in grain samples from Lamwo and Kitgum in households with NS and without NS. [file 13104_2018_3774_MOESM1_ESM.docx]

**Table S1.** Concentration of mycotoxins in grain samples from Lamwo and Kitgum in households with NS and without NS

| **District** | **Village** | **Household** | **Sample** | **Number of Nodding syndrome Cases** | **Total Aflatoxins** | **Ochratoxins** | **DON** |
| --- | --- | --- | --- | --- | --- | --- | --- |
| Kitgum | Lamit Tumangu | HH1 | Millet | 1 | 0.000 | 1.647 | 0.000 |
| Lamwo | Beyogoya | HH2 | Sorghum | 1 | 10.110 | 6.125 | 0.000 |
| Lamwo | Beyogoya | HH3 | Millet | 1 | 0.000 | 3.158 | 0.000 |
| Kitgum | Lamit Tumangu | HH4 | Sorghum | 1 | 16.201 | 1.415 | 0.375 |
| Lamwo | Apyeta West | HH5 | Sorghum | 1 | 0.000 | 0.685 | 0.000 |
| Kitgum | Lamit Tumangu | HH6 | Sorghum | 3 | 10.923 | 2.637 | 0.000 |
| Lamwo | Apyeta West | HH7 | Sorghum | 3 | 5.897 | 2.049 | 0.000 |
| Kitgum | Lamit Tumangu | HH8 | Millet | 3 | 7.845 | 0.416 | 1.904 |
| Lamwo | Apyeta West | HH9 | Sorghum | 1 | 11.149 | 1.193 | 0.481 |
| Lamwo | Beyogoya | HH10 | Sorghum | 2 | 13.918 | 3.661 | 0.551 |
| Kitgum | Lamit Tumangu | HH11 | Sorghum | 1 | 13.321 | 1.568 | 0.000 |
| Kitgum | Lamit Tumangu | HH12 | Sorghum | 3 | 26.106 | 7.647 | 1.138 |
| Kitgum | Lamit Tumangu | HH13 | Maize | 1 | 3.449 | 0.000 | 0.695 |
| Lamwo | Beyogoya | HH14 | Sun flower | 1 | 5.348 | 0.000 | 0.615 |
| Lamwo | Apyeta South | HH15 | Sorghum | 1 | 9.170 | 3.007 | 0.456 |
| Kitgum | Lamit Tumangu | HH16 | Sorghum | 1 | 8.516 | 3.501 | 0.573 |
| Lamwo | Beyogoya | HH17 | Millet | 1 | 3.380 | 0.322 | 0.000 |
| Kitgum | Lamit Tumangu | HH18 | Maize | 2 | 4.786 | 0.505 | 1.170 |
| Kitgum | Lamit Tumangu | HH19 | Sorghum | 1 | 68.232 | 3.687 | 0.788 |
| Lamwo | Beyogoya | HH20 | Millet | 2 | 2.881 | 0.000 | 0.000 |
| Kitgum | Lamit Tumangu | HH21 | Sorghum | 2 | 14.412 | 3.627 | 0.000 |
| Kitgum | Lamit Tumangu | HH22 | Sorghum | 2 | 9.103 | 2.713 | 1.358 |
| Kitgum | Lamit Tumangu | HH23 | Maize | 1 | 1.091 | 0.505 | 2.606 |
| Kigum | Lamit Tumangu | HH24 | Sorghum | 3 | 4.139 | 4.124 | 0.000 |
| Kitgum | Okidi Central | HH25 | Sorghum | 1 | 0.000 | 3.203 | 0.000 |
| Kitgum | Okidi Central | HH26 | Sorghum | 2 | 4.384 | 1.382 | 0.000 |
| Lamwo | Apyeta West | HH27 | Sorghum | 1 | 5.869 | 0.000 | 0.000 |
| Kitgum | Lamit Tumangu | HH28 | Maize | 1 | 8.146 | 1.233 | 0.000 |
| Kitgum | Okidi Central | HH29 | Millet | 2 | 0.000 | 1.716 | 0.050 |
| Kitgum | Okidi Central | HH30 | Sesame | 1 | 0.000 | 1.817 | 0.000 |
| Kitgum | Lamit Tumangu | HH31 | Sesame | 1 | 0.000 | 2.627 | 0.003 |
| Kitgum | Lamit Tumangu | HH32 | Sesame | 1 | 0.000 | 1.205 | 0.000 |
| Kitgum | Lamit Tumangu | HH33 | Sesame | 1 | 0.000 | 1.967 | 0.336 |
| Kitgum | Okidi Cenral | HH34 | Sesame | 2 | 1.599 | 0.000 | 0.259 |
| Kitgum | Lamit Tumangu | HH35 | Cheer | 3 | 2.702 | 2.459 | 0.792 |
| Kitgum | Lamit Tumangu | HH36 | Sesame | 2 | 0.000 | 1.903 | 0.190 |
| Kitgum | Lamit Tumangu | HH37 | Cheer | 2 | 0.000 | 1.059 | 0.296 |
| Kitgum | Lamit Tumangu | HH38 | Cheer | 3 | 0.669 | 1.198 | 0.468 |
| Lamwo | Apyeta South | HN1 | Sorghum | 0 | 3.843 | 0.056 | 0.000 |
| Lamwo | Beyogoya | HN2 | Ground nuts | 0 | 3.678 | 1.077 | 0.000 |
| Lamwo | Beyogoya | HN3 | Sorghum | 0 | 7.770 | 1.663 | 0.000 |
| Lamwo | Beyogoya | HN4 | Millet | 0 | 14.811 | 1.050 | 0.000 |
| Lamwo | Apyeta South | HN5 | Sorghum | 0 | 13.037 | 3.610 | 0.000 |
| Lamwo | Beyogoya | HN6 | Millet | 0 | 14.321 | 0.939 | 0.000 |
| Lamwo | Abam | HN7 | Sorghum | 0 | 5.031 | 3.435 | 0.000 |
| Lamwo | Abam | HN8 | Sorghum | 0 | 4.639 | 2.120 | 1.342 |
| Lamwo | Abam | HN9 | Sorghum | 0 | 6.016 | 1.322 | 1.217 |
| Kitgum | Okidi Central | HN10 | Millet | 0 | 3.374 | 0.248 | 0.000 |
| Lamwo | Apyeta South | HN11 | Sorghum | 0 | 12.928 | 6.365 | 0.000 |
| Kitgum | Okidi Central | HN12 | Maize | 0 | 2.092 | 0.359 | 0.276 |
| Lamwo | Abam | HN13 | Maize | 0 | 1.835 | 0.420 | 0.000 |
| Lamwo | Apyeta South | HN14 | Sorghum | 0 | 13.059 | 6.322 | 1.467 |
| Lamwo | Beyogoya | HN15 | Sorghum | 0 | 8.643 | 3.386 | 0.505 |
| Lamwo | Abam | HN16 | Maize | 0 | 4.574 | 0.000 | 0.000 |
| Kitgum | Okidi Central | HN17 | Maize | 0 | 5.336 | 0.000 | 0.676 |
| Lamwo | Abam | HN18 | Maize | 0 | 4.238 | 0.596 | 0.000 |
| Lamwo | Apyeta South | HN19 | Sorghum | 0 | 14.386 | 4.578 | 1.625 |
| Lamwo | Beyogoya | HN20 | Sorghum | 0 | 8.452 | 3.837 | 1.738 |
| Lamwo | Beyogoya | HN21 | Sorghum | 0 | 6.787 | 3.498 | 0.000 |
| Lamwo | Beyogoya | HN22 | Sorghum | 0 | 8.510 | 5.045 | 0.000 |
| Lamwo | Beyogoya | HN23 | Sorghum | 0 | 12.348 | 7.424 | 0.000 |
| Lamwo | Beyogoya | HN24 | Sorghum | 0 | 0.000 | 0.000 | 0.000 |
| Lamwo | Beyogoya | HN25 | Sorghum | 0 | 4.372 | 4.643 | 0.000 |
| Lamwo | Beyogoya | HN26 | Sorghum | 0 | 15.608 | 5.647 | 0.000 |
| Lamwo | Beyogoya | HN27 | Sorghum | 0 | 5.454 | 3.732 | 0.000 |
| Lamwo | Beyogoya | HN28 | Sorghum | 0 | 0.000 | 0.458 | 0.000 |
| Lamwo | Beyogoya | HN29 | Sorghum | 0 | 0.000 | 0.208 | 0.017 |
| Lamwo | Abam | HN30 | Millet | 0 | 0.000 | 1.801 | 0.000 |
| Lamwo | Beyogoya | HN31 | Sesame | 0 | 0.000 | 0.000 | 0.001 |
| Lamwo | Beyogoya | HN32 | Sesame | 0 | 0.000 | 0.094 | 0.013 |
| Lamwo | Beyogoya | HN33 | Sesame | 0 | 0.000 | 2.745 | 0.013 |
| Lamwo | Beyogoya | HN34 | Sesame | 0 | 0.000 | 2.574 | 0.159 |
| Lamwo | Beyogoya | HN35 | Sesame | 0 | 0.000 | 0.000 | 0.374 |
| Lamwo | Beyogoya | HN36 | Sesame | 0 | 0.000 | 0.289 | 0.407 |
| Lamwo | Beyogoya | HN37 | Sesame | 0 | 0.000 | 2.096 | 0.424 |
| Lamwo | Beyogoya | HN38 | Sesame | 0 | 0.000 | 3.124 | 0.512 |
| Lamwo | Beyogoya | HN39 | Sesame | 0 | 0.000 | 2.477 | 0.941 |
| Lamwo | Beyogoya | HN40 | Sesame | 0 | 3.249 | 1.983 | 0.427 |
| Kitgum | Lamit Tumangu | HN41 | Sesame | 0 | 3.734 | 1.226 | 0.154 |
| Kitgum | Lamit Tumangu | HN42 | Sesame | 0 | 0.000 | 0.838 | 0.044 |
| Kitgum | Lamit Tumangu | HN43 | Sesame | 0 | 0.000 | 0.496 | 0.741 |
| Lamwo | Abam | HN44 | Sesame | 0 | 0.000 | 2.556 | 0.123 |
| Lamwo | Abam | HN45 | Sesame | 0 | 2.254 | 1.716 | 0.955 |
| Lamwo | Abam | HN46 | Sesame | 0 | 4.456 | 0.000 | 0.825 |
