# Supplementary material for: Is nodding syndrome in northern Uganda linked to consumption of mycotoxin contaminated food grains?
Source: BMC Res Notes. 2018 Sep 24;11:678. doi: 10.1186/s13104-018-3774-y (PMC6154931; doi:10.1186/s13104-018-3774-y)
Supplement: Supplementary file 2 — Additional file 2: Table S2. Sampling sites for cereal grains. [file 13104_2018_3774_MOESM2_ESM.docx]

**Table S2.** Sampling sites for cereal grains.

| **District** | **Village** | **No. of households visited** | **No. of food grain samples collected** |
| --- | --- | --- | --- |
| Kitgum | Okidi Central | 8 | 11 |
|  | Lamit Tumangu | 25 | 26 |
| Lamwo | Beyogoya | 31 | 29 |
|  | Apeyta South | 6 | 11 |
|  | Apeyta West | 4 | 5 |
|  | Abam | 10 | 21 |
|  |  |  |  |
| **Total** |  | **84** | **103** |
